# Epstein–Barr Virus DNA Detection Without Detectable Expression of Selected Viral Transcripts in Pediatric Patients with Systemic Lupus Erythematosus

**DOI:** 10.3390/genes17090988

**Published:** 2026-08-24

**Authors:** Ilaria Galliano, Aurora Rotondaro, Anna Massobrio, Anna Clemente, Alessia Cito, Cristina Calvi, Stefano Gambarino, Francesco Licciardi, Massimiliano Bergallo

**Affiliations:** 1 Department of Public Health and Pediatric Sciences, University of Turin, Piazza Polonia 94, 10126 Turin, Italy; aurora.rotondaro@edu.unito.it (A.R.); anna.clemente@unito.it (A.C.); cristina.calvi@unito.it (C.C.); gambarino.stefano@gmail.com (S.G.); massimiliano.bergallo@unito.it (M.B.); 2 Pediatric Laboratory, Department of Children’s Pathology and Care, Regina Margherita Children’s Hospital, Piazza Polonia 94, 10126 Turin, Italy; 3 Laboratory Medicine, Regina Margherita Children’s Hospital, 10126 Turin, Italy; amassobrio@cittadellasalute.to.it (A.M.); alessia.cito@edu.unito.it (A.C.); 4 Immunorheumatology Unit, Regina Margherita Children’s Hospital, University Hospital City of Health and Sciences of Turin, 10126 Turin, Italy; francesco.licciardi@gmail.com

**Keywords:** Epstein-Barr virus, systemic lupus erythematosus, EBV DNA, *LMP1*, *NFKB1*, *IL-10*, *TLR3*, apoptosis, caspase-3

## Abstract

Background: Epstein–Barr virus (EBV) has long been linked to systemic lupus erythematosus (SLE), mainly through molecular mimicry, altered B-cell biology, defective antiviral immune surveillance and intermittent viral reactivation. However, the detection of EBV DNA in clinical samples does not necessarily indicate transcriptionally active infection or direct viral modulation of host pathways. Methods: Peripheral blood samples from 39 pediatric patients with SLE and 29 healthy controls were analyzed for host immune and apoptotic gene expression, including *NFKB1*, *IL10*, *TLR3*, *BCL2*, *BAX* and *CASP3*. EBV DNA was assessed in both patients and controls. In EBV DNA-positive SLE samples, the expression of EBV transcripts representative of latent, lytic and immunomodulatory programs, including *LMP1*, *EBNA1*, *BZLF1*, and viral *IL-10*, was also evaluated. Host gene expression was additionally compared between EBV DNA-positive and EBV DNA-negative patients with SLE. Results: EBV DNA was detected in 8 of 39 patients with SLE (20.5%) and in none of the 29 healthy controls (0%; Fisher’s exact test, *p* = 0.0171). None of the EBV DNA-positive samples showed detectable expression of *LMP1*, *EBNA1*, *BZLF1* or viral *IL-10*. Compared with healthy controls, patients with SLE showed increased CASP3 expression that remained significant after FDR correction, whereas the reduction in IL10 expression was nominally significant only in the unadjusted analysis. *NFKB1*, *TLR3*, *BCL2*, and *BAX* did not differ significantly. In the exploratory comparison between EBV DNA-positive and EBV DNA-negative patients with SLE, no host gene remained significantly associated with EBV DNA status after false discovery rate correction. Conclusions: EBV DNA was detected more frequently in patients with SLE than in healthy controls, but this finding was not accompanied by detectable expression of the selected EBV transcripts under the analytical conditions used or by a consistent host transcriptional signature associated with EBV DNA positivity. These findings emphasize the importance of distinguishing EBV DNA detection from detectable viral transcript expression in SLE.

## 1. Introduction

Epstein–Barr virus (EBV) is a ubiquitous human gammaherpesvirus that establishes lifelong persistence in the B-cell compartment after primary infection. Its biology is characterized by a finely regulated alternation between latent and lytic programs, allowing the virus to persist in memory B cells while remaining largely invisible to immune surveillance [1,2,3,4]. Because EBV infects and reprograms B cells, it has attracted particular attention in autoimmune diseases in which B-cell tolerance is altered, including systemic lupus erythematosus (SLE) [5,6,7,8,9].

SLE is a complex systemic autoimmune disease characterized by loss of tolerance to nuclear antigens, production of autoantibodies, immune-complex deposition, complement activation and multi-organ inflammation [10,11]. Genetic predisposition, environmental exposures, hormonal influences and immune dysregulation all contribute to disease onset and progression. Among environmental triggers, EBV is one of the most consistently investigated candidates because of its tropism for B cells and its capacity to shape both innate and adaptive immunity [5,6,7,8,9,12].

Several lines of evidence support an association between EBV and SLE. Patients with SLE show altered antibody responses to EBV antigens, increased viral loads, a higher frequency of EBV-infected B cells, and impaired EBV-specific T-cell control compared with healthy individuals [12,13,14,15,16,17,18,19]. Molecular mimicry between EBV nuclear antigen 1 (*EBNA1*) and lupus autoantigens has also been proposed as a mechanism contributing to autoreactive humoral responses [20,21,22]. More recent studies indicate that EBV may additionally interact with autoimmune susceptibility loci and alter gene regulation in infected B cells [23,24,25,26]. In particular, EBV-derived transcription factors such as *EBNA2* bind multiple autoimmune susceptibility loci, while EBV load-dependent transcriptional changes in B-cell subsets have been associated with SLE genetic risk [23,24]. Together, these observations suggest that the biological effects of EBV in SLE may depend on viral state, host immune control, and the cellular compartment in which the virus persists [1,23,24,25,26].

Among EBV latent genes, latent membrane protein 1 (LMP1) is particularly relevant because it acts as a constitutively active mimic of CD40 and activates NF-κB and other signaling pathways involved in cytokine production and cell survival [27,28,29]. Recent evidence also suggests that LMP1 may influence SLE-related molecular pathways through epigenetic mechanisms; Zhang et al. reported that LMP1 overexpression increased ARID5B expression, enhanced EZH2-mediated H3K27 methylation, and activated NF-κB [30]. EBV also encodes immunomodulatory products, including viral IL-10, which may suppress antiviral immune responses and favor viral persistence [31,32].

Innate immune pathways may also be influenced by EBV. TLR3 and other nucleic-acid sensors participate in antiviral recognition and type I interferon responses, both highly relevant to SLE pathogenesis [33,34]. In parallel, apoptosis and defective clearance of apoptotic material contribute to exposure of nuclear autoantigens and sustained autoimmunity in SLE [35,36,37,38].

Based on these premises, this study aimed to determine whether EBV DNA was more frequently detectable in peripheral blood from pediatric patients with SLE than in healthy controls and to assess the expression of selected EBV transcripts in EBV DNA-positive samples. We also investigated selected host transcripts involved in immune regulation, antiviral sensing, and apoptosis and compared their expression between EBV DNA-positive and EBV DNA-negative patients with SLE. The original working hypothesis was that detectable EBV transcription, particularly *LMP1* expression, would be associated with increased *NFKB1* and *IL10* transcript expression and with a transcriptional pattern characterized by increased *BCL2* and reduced *CASP3* expression.

## 2. Materials and Methods

### 2.1. Study Population

This study enrolled 39 patients with systemic lupus erythematosus (SLE) and 29 control subjects. Patients were recruited from the Immunorheumatology Unit of the Regina Margherita Children’s Hospital in Turin, Italy, where they were regularly followed by pediatric rheumatologists.

The diagnosis of SLE was confirmed by expert clinicians according to internationally accepted classification standards. In particular, patients fulfilled the 2012 Systemic Lupus International Collaborating Clinics (SLICC) criteria, which classify SLE on the basis of combined clinical and immunological features, or on the presence of biopsy-confirmed lupus nephritis together with antinuclear antibodies or anti-double-stranded DNA antibodies.

Cases with incomplete, uncertain, or unverified diagnostic information were not included in the analysis. At the time of sample collection, disease activity was evaluated using the Systemic Lupus Erythematosus Disease Activity Index 2000 (SLEDAI-2K). Demographic, clinical, and laboratory information was obtained retrospectively from medical records.

Healthy controls were recruited from the same hospital setting. They were evaluated for elective reasons, including pre-surgical assessment or diagnostic work-up aimed at excluding genetic, metabolic, endocrinological, or nephrological disorders. At the time of blood sampling, none of the control subjects showed laboratory abnormalities or evidence of active autoimmune, inflammatory, or infectious disease. Healthy controls were independently selected and were not individually matched to patients with SLE for age or sex. Age and sex distributions were subsequently compared between the two groups as described in the Section 2.9.

Whole-blood samples suitable for EBV DNA analysis were available from all 39 patients with SLE and all 29 healthy controls. EBV DNA positivity was therefore evaluated and compared between the two study groups.

### 2.2. Sample Storage

For RNA analysis, 200 μL of whole blood was transferred into a 1.5 mL Eppendorf tube containing 800 μL of RNApro stabilization solution (Biomole, Turin, Italy). The mixture was thoroughly homogenized by vortexing to ensure adequate sample preservation. Samples were then frozen and maintained at −80 °C until RNA extraction.

For EBV DNA analysis, an additional aliquot of 300 μL of untreated whole blood was collected from each patient with SLE and each healthy control, stored at −20 °C, and subsequently processed for genomic DNA extraction using the Maxwell automated platform and the appropriate Promega extraction protocol.

### 2.3. Total RNA Extraction

Total RNA was isolated from whole-blood samples using the Maxwell automated extraction system (Promega, Madison, WI, USA) together with the RNA Blood Kit, which incorporates a DNase treatment step to minimize genomic DNA carryover. RNA yield and quality were evaluated by UV spectrophotometry, measuring absorbance at 260 and 280 nm. Quantification was performed according to the Beer–Lambert principle.

RNA concentration was further verified using a NanoDrop ND-1000 spectrophotometer (Thermo Fisher Scientific, Waltham, MA, USA). For each measurement, 1 μL of RNA was analyzed at room temperature. Samples showing an A260/A280 ratio between 1.8 and 2.1 were considered of suitable purity for downstream applications. To exclude residual genomic DNA contamination, RNA extracts were subjected to amplification in the absence of reverse transcription. Purified RNA was stored at −80 °C until further use.

### 2.4. Reverse Transcription

Complementary DNA was synthesized from 400 ng of total RNA using the ImProm-II reverse-transcription system (Promega, Madison, WI, USA). Each 20 μL reaction contained 2 μL of 10× buffer, 4.8 μL of MgCl_2_ 25 mM, 2 μL of ImProm-II reverse transcriptase, 1 μL of RNase inhibitor 20 U/μL, 0.4 μL of random hexamers 250 μM, 2 μL of mixed dNTPs 100 mM, and nuclease-free water to final volume.

Reverse transcription was performed in a GeneAmp PCR System 9700 thermal cycler (Applied Biosystems, Foster City, CA, USA). The reaction protocol consisted of primer annealing at 25 °C for 5 min, cDNA synthesis at 42 °C for 60 min, and enzyme inactivation at 70 °C for 15 min. The resulting cDNA samples were stored at −80 °C until analysis. All procedures were carried out in accordance with the manufacturers’ instructions. As a control for genomic DNA contamination, RNA samples were also amplified without a reverse-transcription step.

### 2.5. Gene-Expression Analysis by Real-Time PCR

Relative gene expression was evaluated by real-time TaqMan PCR using cDNA obtained from peripheral blood RNA. Host gene-expression analysis included *NFKB1*, *IL10*, *TLR3*, *BCL2*, *BAX*, and *CASP3* in all patients with SLE and healthy controls. In EBV DNA-positive SLE samples, the analysis also included selected EBV transcripts associated with latent, lytic, and immunomodulatory viral programs, namely *LMP1*, *EBNA1*, *BZLF1*, and viral *IL-10*.

Amplification reactions were performed on the ABI PRISM 7500 real-time PCR system (Life Technologies, Austin, TX, USA). Each reaction was carried out in a final volume of 20 μL, including 4 μL of cDNA and 16 μL of reaction mix prepared with GoTaq Probe Master Mix (Promega, Madison, WI, USA), target-specific primers, hydrolysis probes, and nuclease-free water.

For *IL10*, forward and reverse primers were used at a final concentration of 300 nM each, while the probe was used at 150 nM. *TLR3* amplification was performed with primers at 500 nM and probe at 200 nM. *NFKB1* was analyzed together with *H18S* in a combined reaction. In this assay, *H18S* primers and probe were used at 100 nM, whereas *NFKB1* primers and probe were used at 500 nM and 200 nM, respectively.

The apoptotic markers *BCL2*, *BAX*, and *CASP3* were quantified using a multiplex real-time PCR assay. For each of these targets, forward and reverse primers were added at 500 nM and probes at 200 nM. *H18S* was selected as the endogenous control for normalization. Relative expression levels were calculated after normalization to *H18S* using comparative quantification. The sequences of primers and probes used for host gene-expression analysis, EBV transcript detection, and *BALF5* amplification are reported in Table 1.

### 2.6. EBV Transcript Assay Verification

Before analyzing clinical samples, the real-time PCR assays designed to detect EBV transcripts were verified using a synthetic plasmid control purchased from Eurofins Genomics (Ebersberg, Germany). The plasmid contained the target sequences corresponding to the four EBV transcript assays included in the study: *LMP1*, *EBNA1*, *BZLF1*, and viral *IL-10*.

This plasmid control was used as a positive amplification control to confirm the analytical performance of each EBV transcript assay and to exclude technical failure in reactions yielding negative results in clinical samples.

### 2.7. EBV DNA Extraction and Viral Load Quantification

In addition to RNA-based analyses, genomic DNA was isolated from untreated whole-blood samples obtained from all patients with SLE and healthy controls to evaluate EBV DNA positivity. For this analysis, 300 μL of whole blood, not previously stabilized with RNApro solution, was processed using the Maxwell automated extraction platform (Promega, Madison, WI, USA), following the manufacturer’s protocol.

Purified DNA samples were tested without dilution by real-time PCR targeting the EBV *BALF5* gene. Amplification reactions were carried out in a final volume of 20 μL, composed of 15 μL of reaction mix and 5 μL of DNA template. Each reaction included 10 μL of master mix, *BALF5*-specific forward and reverse primers at a final concentration of 500 nM each, *BALF5*-specific hydrolysis probe at 200 nM, and nuclease-free water to complete the reaction volume.

Absolute EBV DNA quantification was performed using a synthetic BALF5 plasmid standard purchased from Eurofins Genomics (Ebersberg, Germany). The plasmid standard was included in each analytical run to generate the standard curve for viral-load calculation. Viral loads were expressed as EBV DNA copies per milliliter of whole blood.

### 2.8. BALF5 Standard Curve Setup and Analytical Sensitivity

Prior to sample analysis, the EBV *BALF5* real-time PCR assay was optimized using a synthetic BALF5 plasmid standard purchased from Eurofins Genomics (Ebersberg, Germany). Serial plasmid dilutions were prepared to generate the standard curve, corresponding to 5 × 10^1^, 5 × 10^2^, 5 × 10^3^, 5 × 10^4^, 5 × 10^5^, and 5 × 10^6^ copies per reaction.

A lower concentration of 5 copies per reaction was tested in five replicate reactions to evaluate detection at concentrations below the linear quantification range. The lowest standard point included in the linear quantification range was 50 copies per reaction, and assay reproducibility at this concentration was evaluated by replicate testing.

Clinical samples generating a clear amplification curve below the lower boundary of the linear quantification range were retested in triplicate. Samples showing reproducible amplification in all replicate reactions were classified as EBV DNA-positive but below the LLOQ, and no quantitative viral-load value was assigned.

### 2.9. Statistical Analysis

Categorical variables were reported as absolute numbers and percentages. The frequency of EBV DNA positivity in patients with SLE and healthy controls was compared using Fisher’s exact test because of the presence of a zero count in the healthy-control group. Continuous variables were summarized as median and interquartile range (IQR, 25th–75th percentile). Gene-expression data were analyzed as relative quantification values after normalization to the endogenous reference gene. Age distributions between patients with SLE and healthy controls were compared using the Mann–Whitney U test, whereas sex distributions were compared using Fisher’s exact test. Comparisons between patients with SLE and healthy controls were performed using the Mann–Whitney U test. Within the SLE group, expression levels of *NFKB1*, *IL10*, *TLR3*, *BCL2*, *BAX*, and *CASP3* were compared between EBV DNA-positive and EBV DNA-negative patients using the Mann–Whitney U test. Clinical and hematological variables were also compared between EBV DNA-positive and EBV DNA-negative patients with SLE. Continuous variables were analyzed using the Mann–Whitney U test, whereas categorical variables were compared using Fisher’s exact test. Because six host genes were evaluated in both the SLE-versus-healthy-control comparison and the EBV DNA-positive versus EBV DNA-negative subgroup analysis, the false discovery rate was controlled at 5% using the two-stage linear step-up procedure of Benjamini, Krieger, and Yekutieli. Both unadjusted *p*-values and FDR-adjusted *p*-values were considered in the interpretation of the results. An unadjusted *p*-value < 0.05 was considered nominally significant, whereas significance after multiple-testing correction was determined according to the 5% false discovery rate threshold. Statistical analyses and graph generation were performed using Prism software (Version 7, GraphPad Software, La Jolla, CA, USA).

## 3. Results

### 3.1. Clinical and Demographic Characteristics of the Study Population

The demographic and clinical features of the study population are summarized in Table 2. The cohort included 39 patients with SLE and 29 healthy controls. Female participants represented the majority in both groups, accounting for 82% of SLE patients and 76% of controls. The median age at sampling was 13.9 years in the SLE group and 13.0 years in the control group. Age and sex distributions were formally compared between patients with SLE and healthy controls. No statistically significant differences were observed for age (Mann–Whitney U test, *p* = 0.2018) or sex distribution (Fisher’s exact test, *p* = 0.5586).

Among patients with SLE, 8 subjects (21%) were sampled at disease onset, whereas the median disease duration in the overall SLE cohort was 11.1 months. At blood collection, disease activity was generally low to moderate, with a median SLEDAI-2K score of 4. Ongoing treatment was recorded in 30 patients (77%), most commonly hydroxychloroquine, followed by oral prednisone and mycophenolate mofetil.

### 3.2. Expression of Host Immune and Apoptotic Markers

**Figure 1:**
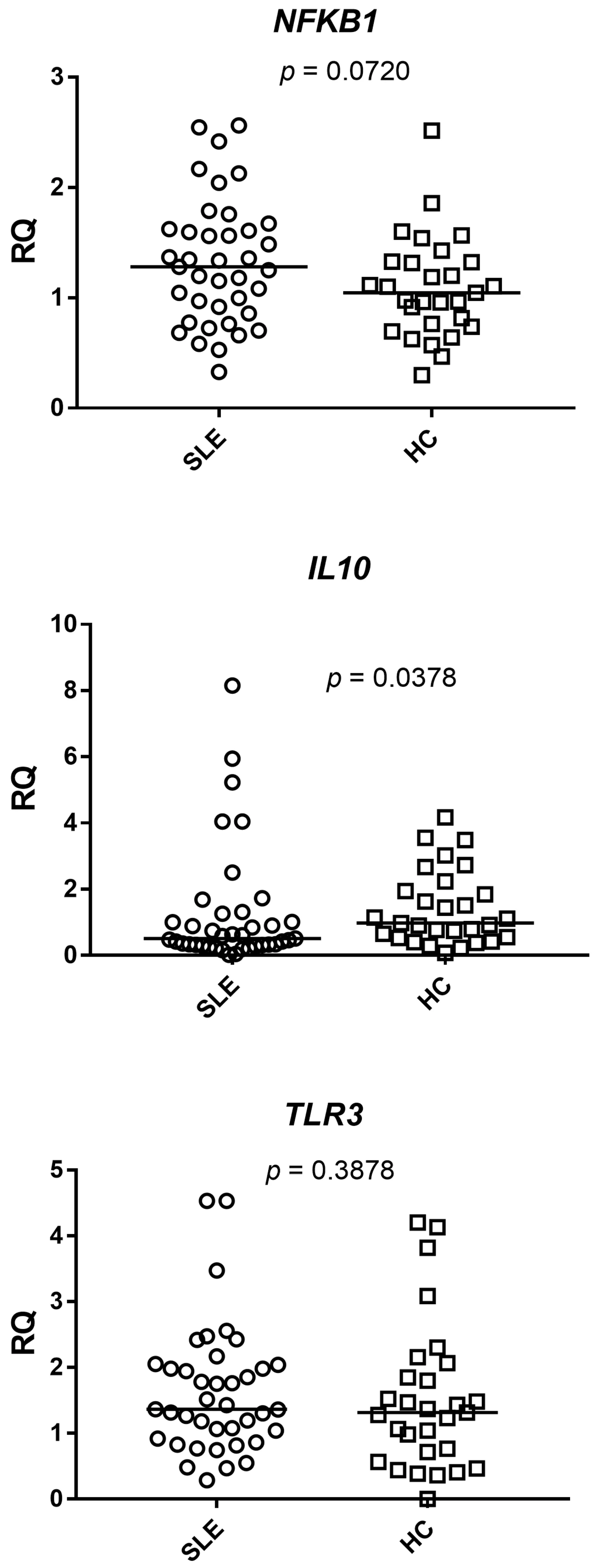
Expression of immune-related host transcripts in SLE patients and healthy controls. Relative expression levels of *NFKB1*, *IL10*, and *TLR3* were assessed by real-time PCR and expressed as relative quantification (RQ). Each symbol represents an individual sample, and horizontal lines indicate median values. The *p*-values displayed in the panels are unadjusted; FDR-adjusted *p*-values are reported in the Results. Patients with SLE showed a non-significant trend toward higher *NFKB1* expression, nominally reduced *IL10* expression in the unadjusted analysis, which did not remain significant after FDR correction, and unchanged *TLR3* expression compared with healthy controls.

The expression of selected host transcripts involved in immune regulation, innate antiviral sensing, and apoptosis was compared between patients with SLE and healthy controls. Relative quantification analysis showed a trend toward increased *NFKB1* expression in SLE samples compared with controls; however, this difference did not reach statistical significance (unadjusted *p* = 0.0720; FDR-adjusted *p* = 0.0845). *IL10* expression was lower in the SLE group than in healthy controls and reached nominal significance in the unadjusted analysis (*p* = 0.0378), but this difference did not remain significant after FDR correction (FDR-adjusted *p* = 0.0845). No significant difference was observed for *TLR3* expression between the two groups (unadjusted *p* = 0.3878; FDR-adjusted *p* = 0.3393) (Figure 1).

For immune-related transcripts, median expression values (IQR 25–75%) in SLE patients and healthy controls were, respectively, 1.28 (0.89–1.62) and 0.93 (0.76–1.30) for NFKB1, 0.52 (0.32–1.14) and 0.98 (0.56–1.95) for IL10, and 1.36 (0.98–2.01) and 1.32 (0.71–1.85) for *TLR3*.

**Figure 2:**
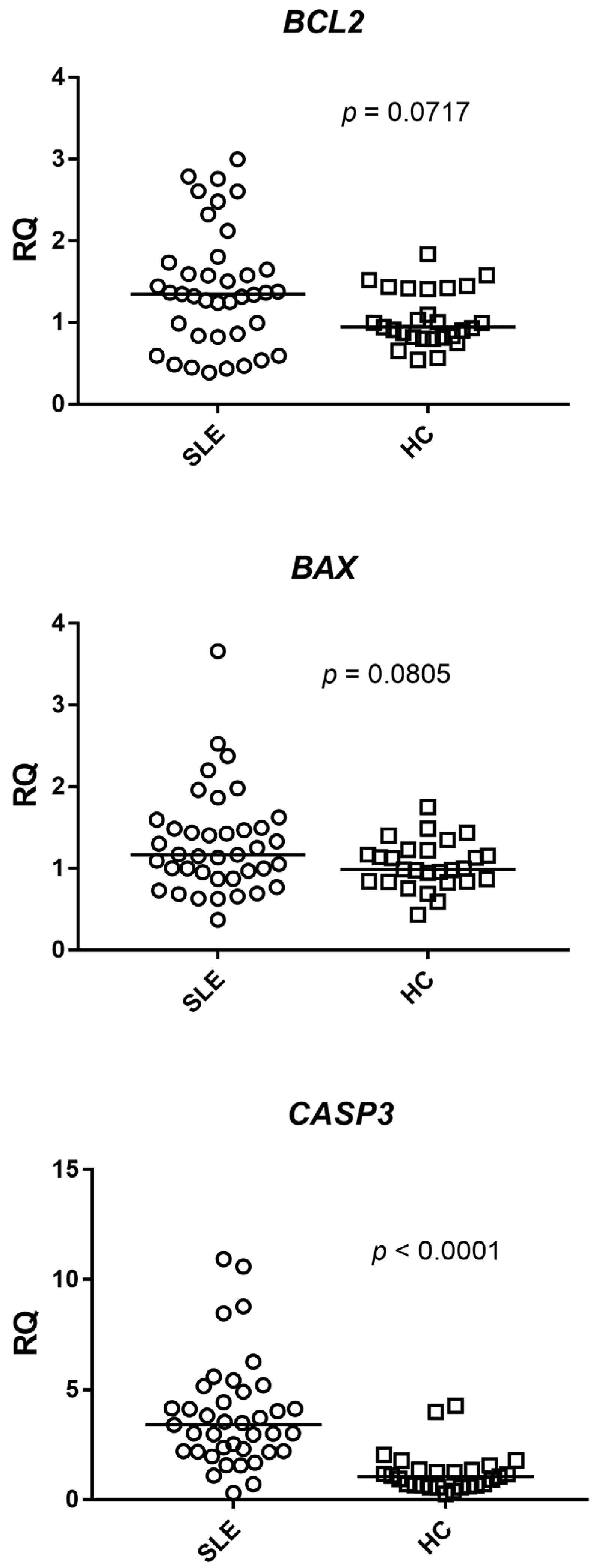
Expression of apoptosis-related host transcripts in SLE patients and healthy controls. Relative expression levels of *BCL2*, *BAX*, and *CASP3* were evaluated by real-time PCR and expressed as relative quantification (RQ). Each symbol represents an individual sample, and horizontal lines indicate median values. The *p*-values displayed in the panels are unadjusted; FDR-adjusted *p*-values are reported in the Results. *BCL2* and *BAX* showed non-significant upward trends in SLE samples, whereas CASP3 expression remained significantly increased after FDR correction in patients with SLE compared with healthy controls.

The analysis of apoptosis-related genes showed a non-significant tendency toward higher *BCL2* expression in SLE patients compared with controls (unadjusted *p* = 0.0717; FDR-adjusted *p* = 0.0845). A similar non-significant trend was observed for *BAX* (unadjusted *p* = 0.0805; FDR-adjusted *p* = 0.0845). In contrast, *CASP3* expression was markedly increased in SLE samples and remained statistically significant after FDR correction, showing a highly significant difference compared with healthy controls (unadjusted *p* < 0.0001; FDR-adjusted *p* = 0.0005) (Figure 2).

For apoptosis-related genes, median expression values (IQR 25–75%) in SLE patients and healthy controls were, respectively, 1.35 (0.85–1.69) and 0.94 (0.82–1.41) for BCL2, 1.16 (0.91–1.49) and 0.99 (0.84–1.19) for BAX, and 3.40 (2.21–4.67) and 1.06 (0.66–1.35) for *CASP3*.

Overall, these data indicate that the most robust host transcriptional difference between patients with SLE and healthy controls was the increase in CASP3 expression. The reduction in IL10 expression reached nominal significance in the unadjusted analysis but did not remain significant after FDR correction, whereas the remaining transcripts showed no statistically significant differences after multiple-testing correction.

### 3.3. BALF5 Assay Performance and EBV DNA Prevalence

The plasmid-based *BALF5* standard curve showed linear amplification over the tested dilution range, with 50 copies per reaction representing the lowest point of the linear quantification interval. The assay showed acceptable reproducibility at this concentration, with a coefficient of variation below the predefined 5% threshold. The lowest tested concentration was 5 copies per reaction. At this concentration, amplification was observed in 2 out of 5 replicates, corresponding to a detection rate of 40%. Therefore, 5 copies per reaction represented the lowest concentration at which occasional amplification was observed, but it was not included in the linear quantification range.

EBV DNA was detected in 8 out of 39 patients with SLE (20.5%) and in none of the 29 healthy controls (0%) (Table 3). The difference in EBV DNA positivity between the two groups was statistically significant (Fisher’s exact test, *p* = 0.0171).

The difference in EBV DNA positivity between the two groups was assessed using Fisher’s exact test (*p* = 0.0171).

Among EBV DNA-positive SLE samples, quantifiable viral loads ranged from approximately 1.3 × 10^3^ to 6.0 × 10^4^ copies/mL of whole blood. Two positive samples showed detectable amplification below the lower limit of linear quantification (Table 4). Both samples were retested in triplicate and showed reproducible amplification in all replicate reactions, consistently below the linear quantification range. They were therefore classified as EBV DNA-positive but below the LLOQ, and no quantitative viral-load value was assigned.

### 3.4. Absence of Detectable Selected EBV Transcripts in EBV DNA-Positive SLE Samples

All EBV transcript assays were successfully verified using the synthetic plasmid control, confirming adequate amplification of *LMP1*, *EBNA1*, *BZLF1*, and viral *IL-10* target sequences. None of these transcripts was detected in the eight EBV DNA-positive SLE samples. Thus, EBV DNA detection was not accompanied by measurable expression of the selected latent, lytic, or immunomodulatory viral genes.

### 3.5. Host Gene Expression According to EBV DNA Status in Patients with SLE

To assess whether EBV DNA-positive and EBV DNA-negative patients differed in major clinical or hematological characteristics that could influence whole-blood gene-expression profiles, the two groups were compared for disease activity, disease duration, anti-dsDNA levels, disease-onset status, ongoing treatment, and peripheral blood leukocyte counts. No statistically significant differences were observed for any of the variables examined (Table 5).

**Figure 3:**
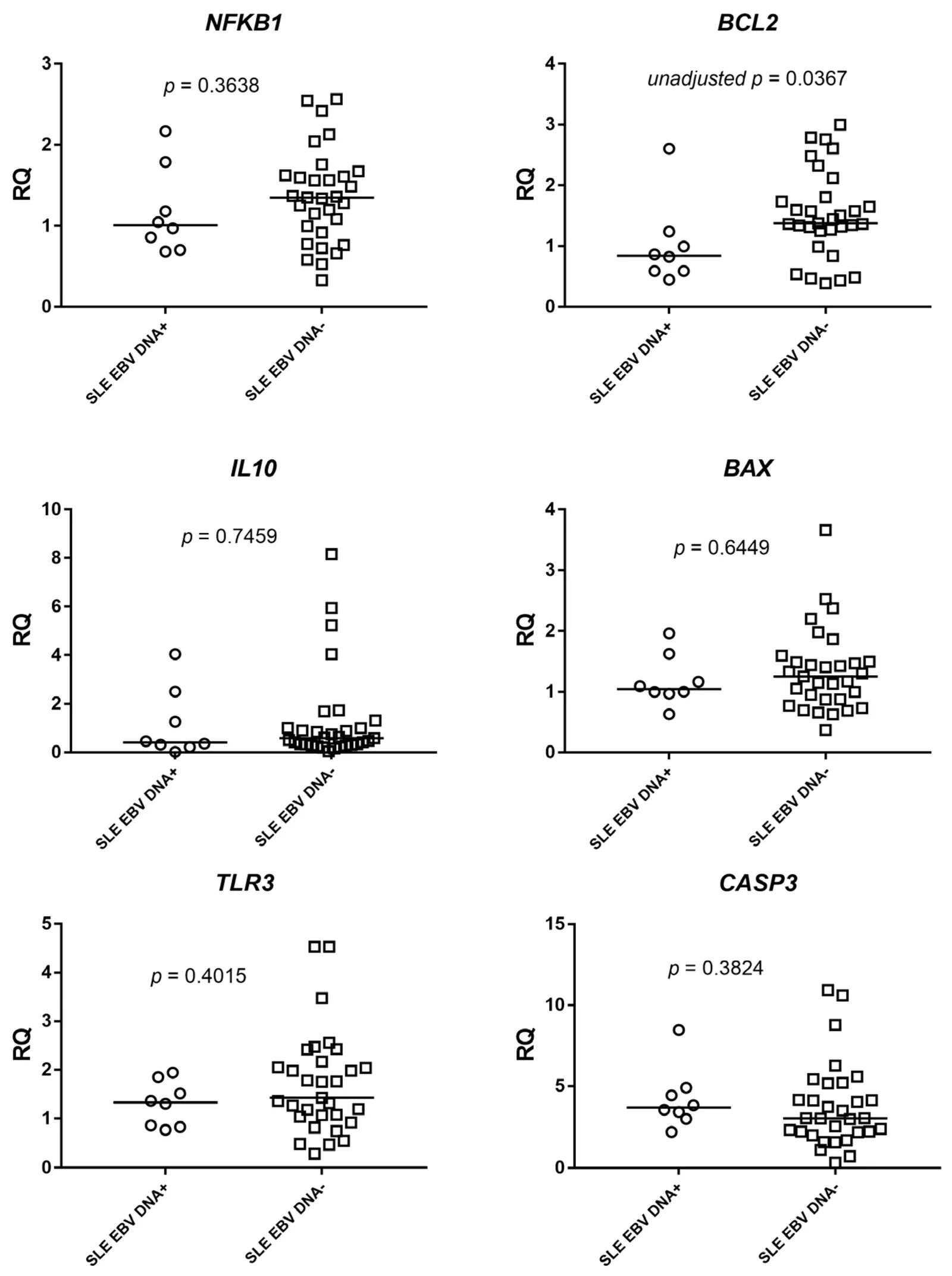
Host gene expression according to EBV DNA status in patients with systemic lupus erythematosus. Relative expression levels of *NFKB1*, *IL10*, *TLR3*, *BCL2*, *BAX*, and *CASP3* were compared between EBV DNA-positive patients with SLE (SLE EBV DNA+, *n* = 8) and EBV DNA-negative patients with SLE (SLE EBV DNA−, *n* = 31). Each symbol represents an individual patient, and horizontal lines indicate median values. The *p*-values displayed in the panels are unadjusted and were obtained using the Mann–Whitney U test. *BCL2* reached nominal significance in the unadjusted analysis (*p* = 0.0367), but no comparison was identified as a discovery after false discovery rate correction (FDR-adjusted *p* = 0.2312).

To investigate whether EBV DNA positivity was associated with a distinct host transcriptional profile, patients with SLE were stratified into EBV DNA-positive (*n* = 8) and EBV DNA-negative (*n* = 31) groups. No nominally significant differences were observed in *NFKB1*, *IL10*, *TLR3*, *BAX*, or *CASP3* expression between the two groups, with unadjusted *p*-values of 0.3638, 0.7459, 0.4015, 0.6449, and 0.3824, respectively (Figure 3).

*BCL2* expression was lower in EBV DNA-positive than in EBV DNA-negative patients and reached nominal significance in the unadjusted analysis (*p* = 0.0367) (Figure 3). However, after controlling the false discovery rate at 5% using the two-stage linear step-up procedure of Benjamini, Krieger, and Yekutieli, no comparison remained statistically significant. The FDR-adjusted *p*-value for *BCL2* was 0.2312. Overall, EBV DNA positivity was not associated with a statistically robust host transcriptional signature involving the investigated immune-regulatory or apoptosis-related genes.

To assess potential clinical and hematological differences between the two SLE subgroups, additional comparisons were performed according to EBV DNA status. No statistically significant differences were observed between EBV DNA-positive and EBV DNA-negative patients with respect to SLEDAI-2K score, disease duration, anti-dsDNA levels, disease-onset status, or ongoing treatment. Similarly, total white blood cell, neutrophil, and lymphocyte counts did not differ significantly between the two groups.

## 4. Discussion

The present study provides three principal findings. First, EBV DNA was detected in 8 of 39 patients with SLE (20.5%) but in none of the 29 healthy controls, resulting in a statistically significant difference between the two groups. Second, none of the EBV DNA-positive SLE samples showed detectable expression of the selected latent, lytic, or immunomodulatory EBV transcripts, including *LMP1*, *EBNA1*, *BZLF1*, and viral *IL-10*. Third, the exploratory comparison between EBV DNA-positive and EBV DNA-negative patients with SLE did not identify a statistically robust host transcriptional profile associated with EBV DNA status. Together, these findings indicate that, although circulating EBV DNA was more frequently detectable in patients with SLE, its presence was not accompanied by evidence of a detectable expression of the selected EBV transcripts or by the expected EBV-related host transcriptional pattern.

The absence of detectable EBV DNA in the healthy-control group strengthens the observed difference between patients with SLE and controls under the analytical conditions used in this study. Nevertheless, this finding should not be interpreted as evidence that the healthy controls had never been infected with EBV or lacked latent viral persistence. EBV establishes lifelong latent infection in a large proportion of the population, predominantly within rare memory B cells, and its detection in whole blood depends on viral burden, cellular composition, sample type, age, and assay sensitivity. Therefore, the present results indicate a difference in detectable circulating EBV DNA rather than a difference in previous exposure to EBV.

This distinction is biologically important. The literature on EBV and SLE demonstrates a strong epidemiological and mechanistic association, but it also emphasizes heterogeneity in viral state and host control [1,5,6,7,8,9,12,13,14,15,16,17,18,19]. Detection of EBV DNA alone cannot distinguish between latent carriage, low-level viral persistence, abortive infection, cell-free viral DNA or active transcriptional programs. Therefore, our findings support the view that EBV DNA positivity in SLE should be interpreted cautiously and should not automatically be equated with transcriptionally active infection or direct viral immunomodulation [1,6,13,26].

Our results are particularly relevant when interpreted in light of recent transcriptomic studies. Akutsu et al. demonstrated that increasing EBV load is associated with profound transcriptional alterations in B-cell subsets and with enrichment of SLE-associated genetic-risk loci [24]. Their data support a model in which EBV load can influence host gene regulation, especially in B-cell compartments, through interactions between viral transcriptional regulators and the genetic architecture of immune-mediated diseases [24]. In contrast, despite detectable EBV DNA in a subset of our patients, no viral transcripts were identified. This finding indicates that expression of the selected EBV transcripts was not detectable in the analyzed bulk peripheral blood samples, despite the presence of detectable EBV DNA.

The absence of detectable *LMP1* expression is central to the interpretation of the host gene-expression findings. LMP1 is one of the main EBV proteins capable of activating NF-kB and promoting B-cell survival [28,29,30]. In the original biological model, detectable LMP1 expression was expected to be associated with increased NF-kB and IL-10 expression and with a transcriptional pattern characterized by increased BCL2 and reduced CASP3 expression. However, *NFKB1* expression did not differ significantly between EBV DNA-positive and EBV DNA-negative patients with SLE. The non-significant increase observed in the overall SLE group compared with healthy controls is therefore more likely to reflect the inflammatory background of SLE than a direct EBV-driven signal. SLE itself is characterized by chronic immune activation and involvement of NF-kB-related inflammatory pathways [10,11,33].

The lack of *LMP1* is also noteworthy in comparison with the recent study by Zhang et al., who reported that LMP1 induces NF-kB activation, EZH2 upregulation, H3K27 methylation and increased expression of SLE susceptibility genes such as ARID5B [27]. According to that model, active LMP1 expression could contribute to SLE pathogenesis through both inflammatory and epigenetic mechanisms. In contrast, none of our EBV DNA-positive samples expressed detectable *LMP1* transcripts. Consistent with this observation, NF-kB expression was not significantly associated with EBV DNA status, and no statistically robust LMP1-associated host transcriptional pattern was identified in EBV DNA-positive patients.

The lower host *IL10* expression observed in the overall SLE group reached nominal significance in the unadjusted analysis but did not remain statistically significant after FDR correction. This pattern was nevertheless not consistent with the expression profile expected during an active EBV immunosuppressive program. In settings of EBV-driven immune modulation, both host IL-10 and viral IL-10 may contribute to an environment that favors viral persistence and limits antiviral immunity [31,32]. In the present samples, viral *IL-10* was not detectable in EBV DNA-positive samples and host *IL10* expression did not differ significantly between EBV DNA-positive and EBV DNA-negative patients with SLE. Therefore, the observed tendency toward lower *IL10* expression in SLE compared with healthy controls was not specifically associated with EBV DNA status and should be interpreted cautiously.

*TLR3* expression did not differ between the overall SLE and healthy-control groups and was also not significantly associated with EBV DNA status within the SLE cohort. TLR3 recognizes double-stranded RNA and contributes to antiviral responses [34]. EBV can interact with innate immune pathways, but such effects are likely to depend on viral transcriptional state, cell type and phase of infection [1,33,34]. The absence of TLR3 modulation, together with the lack of detectable *BZLF1* and other selected EBV transcripts, does not support overt lytic reactivation or a strong EBV-dependent alteration of TLR3-mediated antiviral sensing in the analyzed bulk blood samples.

The apoptosis-related profile was the most divergent from the original hypothesis.

*CASP3* expression was markedly increased in the overall SLE group compared with healthy controls, whereas *BCL2* and *BAX* showed only non-significant upward trends. However, *CASP3* and *BAX* expression did not differ significantly between EBV DNA-positive and EBV DNA-negative patients with SLE. *BCL2* expression was nominally lower in EBV DNA-positive patients in the unadjusted analysis, but this difference did not remain significant after false discovery rate correction. Therefore, the *BCL2* result should be regarded as exploratory rather than as evidence of a definitive association. Moreover, the direction of the nominal difference was opposite to that expected under the original hypothesis, which predicted higher *BCL2* transcript expression in association with EBV-related LMP1 activity. Overall, these findings do not support the expected LMP1-associated host transcriptional pattern. The marked increase in *CASP3* transcript expression observed in the overall SLE cohort may be relevant in the context of the established involvement of apoptosis in SLE pathogenesis [35,36,37,38]; however, transcript abundance alone does not provide evidence of increased caspase-3 activity or apoptosis.

Taken together, the host gene-expression findings suggest that the dominant biological signal in these samples was related to the autoimmune and apoptotic background of SLE rather than to detectable circulating EBV DNA. In particular, the significant increase in *CASP3* and the nominal reduction in *IL10* observed in the overall SLE group were not specifically associated with EBV DNA status. The absence of detectable LMP1 expression and the lack of increased BCL2 expression further argue against the expected LMP1-associated host transcriptional pattern in the analyzed samples.

This interpretation also helps reconcile the present data with previous studies showing EBV reactivation, increased viral load or defective EBV-specific T-cell control in SLE [12,13,14,15,16,17,18,19]. EBV may be relevant in SLE in a context-dependent manner: active during specific disease phases, in selected patients, in particular B-cell subsets or in tissue niches not captured by bulk peripheral blood analysis. Conversely, in other contexts EBV DNA may be detectable without detectable expression of the selected viral transcripts or a dominant host transcriptional effect. Our data are consistent with this latter scenario.

The results also raise methodological considerations. Bulk PCR analysis of peripheral blood may dilute signals from rare infected B-cell subsets. Notably, EBV DNA-positive and EBV DNA-negative patients did not show statistically significant differences in the major clinical and hematological variables examined, including disease activity, disease duration, treatment status, and total leukocyte, neutrophil, and lymphocyte counts. Nevertheless, whole-blood gene-expression measurements are influenced by the relative abundance of different circulating leukocyte populations. Differences in immune-cell composition related to disease status, treatment, infection, or other interindividual factors may therefore have contributed to the observed host gene-expression profiles and cannot be distinguished from cell-intrinsic transcriptional changes in the present analysis. EBV latency programs can be highly restricted, and some viral transcripts may be expressed at levels below detection thresholds. Importantly, the viral transcript panel analyzed in this study was limited to *LMP1*, *EBNA1*, *BZLF1*, and viral *IL-10* and did not include *EBER1* or *EBER2*. Because EBV latency programs are heterogeneous and some restricted latency states may not include LMP1 expression, the absence of these selected transcripts cannot be interpreted as evidence of complete absence of EBV transcription. Moreover, because only peripheral whole-blood samples were analyzed, the absence of detectable viral transcripts in circulating cells does not exclude the presence of EBV-infected cells or transcriptionally active viral reservoirs in tissues or other anatomical compartments not sampled in this study. Rather, it indicates that expression of the selected EBV transcripts was not detectable in the analyzed bulk blood samples under the analytical conditions used. More sensitive approaches, including EBER in situ hybridization, digital PCR, sorted B-cell analysis and single-cell RNA sequencing, could clarify whether rare EBV-positive cells exist with restricted transcriptional activity [1,24,26].

The finding of EBV DNA positivity without viral transcript detection has practical implications for future studies. It suggests that SLE cohorts should be stratified not only by EBV DNA load but also by viral transcriptional state, serological reactivation markers, disease activity, therapy, age, lymphocyte subset distribution and timing relative to disease flares. Longitudinal sampling would be particularly useful, since EBV reactivation may occur intermittently and may follow rather than precede inflammatory flares in some patients [14,17,18]. Future studies should also compare host expression profiles longitudinally within the same patient during EBV DNA-positive and EBV DNA-negative phases, thereby reducing the influence of interindividual variability.

Finally, the present findings should be considered in the context of the ongoing debate on whether EBV is a causal driver of SLE or a disease modifier. Recent Mendelian randomization analyses by Piao et al. failed to demonstrate a direct causal relationship between genetic predictors of SLE and markers of EBV infection [39]. Although this does not exclude a pathogenic role for EBV in specific clinical or cellular contexts, it supports a more nuanced interpretation in which EBV persistence alone may be insufficient to induce the molecular phenotype traditionally attributed to EBV-mediated immune dysregulation. Our data are consistent with this context-dependent model.

This study has several limitations. First, the cohort size was limited, particularly the EBV DNA-positive subgroup, which included only eight patients. The study was based on a retrospectively assembled pediatric SLE cohort for which matched biological material was available for DNA and RNA analyses, and no additional eligible samples with matched DNA and RNA material were available. Consequently, comparisons between EBV DNA-positive and EBV DNA-negative patients were exploratory and had limited statistical power. Second, the cross-sectional design did not allow assessment of intermittent EBV reactivation or temporal relationships between EBV DNA detection, disease activity, and clinical flares. Third, EBV serological data were not available; therefore, the absence of detectable EBV DNA in healthy controls should not be interpreted as absence of previous EBV infection. Fourth, analyses were performed in bulk whole blood, which may dilute signals originating from rare EBV-infected B-cell subsets. Fifth, only four EBV transcripts were investigated, and highly restricted latency programs, expression of other viral genes, or transcript levels below the analytical sensitivity of the assays cannot be excluded. Finally, treatment, disease activity, age, and differences in circulating leukocyte composition may have influenced host gene-expression levels.

Overall, these findings refine rather than refute the EBV-SLE hypothesis. EBV DNA was more frequently detectable in patients with SLE than in healthy controls, but its presence was not accompanied by detectable expression of the selected EBV transcripts or by a statistically robust host transcriptional signature. The major host transcriptional finding observed in the overall SLE cohort was increased CASP3 expression, whereas the reduction in IL10 was nominally significant only before FDR correction; neither finding was specifically associated with EBV DNA positivity. These findings support a context-dependent model in which EBV may contribute to SLE in selected cellular compartments, patient subsets, or disease phases but does not necessarily exert a dominant transcriptional effect in every EBV DNA-positive peripheral blood sample.

## 5. Conclusions

EBV DNA was detected in a subset of patients with SLE but in none of the healthy controls under the analytical conditions used in this study. However, EBV DNA positivity was not accompanied by detectable expression of the selected EBV transcripts analyzed in this study. The expected LMP1-associated transcriptional pattern involving NFKB1, IL10, BCL2, and CASP3 was not observed, and no statistically robust host transcriptional signature was associated with EBV DNA status after false discovery rate correction. These findings indicate that EBV DNA detection in peripheral blood was not accompanied by detectable expression of the selected EBV transcripts under the analytical conditions used. They emphasize the need to distinguish viral DNA detection from detectable viral transcript expression when studying EBV in SLE. This distinction is important for the interpretation of EBV-related biomarker studies and for the design of future studies aimed at identifying patient subsets in whom EBV may have greater biological relevance.

## Figures and Tables

**Table 1:** Primers and probes used for amplification.

Target	Primers		Probes
*H18S*	PF PR	GGGTGGTGGTGCATGG GAGTCTCGTTCGTTATCGGAAT	JOE-TTGGTGGAGCGATTTGTCTGG-BHQ1
*NFKB1*	PF PR	GGCTACACCGAAGCAATTGAA CAGCGAGTGGGCCTGAGA	6FAM-CAGGCAGCCTCCAGCCCAGTGA-BHQ1
*IL-10*	PF PR	ATGAAGGATCAGCTGGACAACTT CCTTGATGTCTGGGTCTTGGT	6FAM-ACCTGGGTTGCCAAGCCTTGTCTG-TAM
*TLR3*	PF PR	TGGTTGGGCCACCTAGAAGTA TCTCCATTCCTGGCCTGTG	6FAM-ACCTGGGCCTTAATGAAATTGGGCAA-TAMRA
*BCL2*	PF PR	TTGGCCCCCGTTGCTT CGGTTATCGTACCCCGTTCTC	HEX-AGCGTGCGCCATCCTTCCCAG-BHQ1
*BAX*	PF PR	GTCGCCCTTTTCTACTTTGCCAG TCCAGCCCAACAGCCGCTCC	T-RED-CCAATGTCCAGCCCATGATGGTTCTGA-BHQ2
*CASP3*	PF PR	TGCGCTGCTCTGCCTTCT CCATGGGTAGCAGCTCCTTC	6FAM-AGCTTCTTCATTTGTGTGCTCCGCTTTCA-TAMRA
*BALF5*	PF PR	GGAATGACGGCGCATTTCTC AGGATGGAAAGGGCATGTGG	6FAM-CCCTCTTGGGCACGCTGGCGCC-BHQ1
*BZLF1*	PF PR	CACACGGAAACCACAACAGC CTTAAACTTGGCCCGGCATT	TRED-CGATACAAGAATCGGGTGGCTTCCAGA-BHQ2
*EBNA1*	PF PR	GAGCCTGACCTGTGATCGTC GGCCATTTCCAGGTCCTGTA	6FAM-CGCCGCGGCCGTCTCCTTTA-BHQ1
*LMP1*	PF PR	CACTTGGAGCCCTTTGTATACTC CCCGAGGATGAACAGCACAA	HEX-ACCCTCCTGCTCATCGCTCTCTGG-BHQ1
Viral *IL-10*	PF PR	CCAGGCCCTGTCAGAAATGA TGGTCTTTGGCTTCAGGGTC	HEX-CCTGGAGGAAGTCATGCCACAGGC-BHQ1

**Table 2:** Demographic and clinical characteristics of SLE patients and HC.

Characteristic	SLE (*n* = 39)	HC (*n* = 29)
Sex (*n*, %)		
Male	7 (18)	7 (24)
Female	32 (82)	22 (76)
Age: median, IQR (25–75%) (years)	13.9, 12.7–15.2	13.0, 10.0–15.9
At diagnosis, *n* (%)	8 (21)	
Disease duration at sampling: median, IQR (25–75%) (months)	11.1, 1.5–26.6	
SLEDAI-2K score at sampling: median, IQR (25–75%)	4, 0–6	
Anti-dsDNA levels, IU/mL: median, IQR (25–75%)	46, 5–176	
Ongoing Treatment (*n*, %)	30 (77)	
Azathioprine	3 (8)	
Hydroxychloroquine	28 (72)	
Oral Prednisone	20 (51)	
Mycophenolate Mofetil	8 (21)	
Anakinra	1 (3)	
Intravenous Methylprednisolone	3 (8)	
Belimumab	2 (5)	
Calcineurin inhibitors (tacrolimus or cyclosporine)	3 (8)	

SLE = Systemic lupus erythematosus; HC = healthy controls; *n* = number; IQR = interquartile range.

**Table 3:** EBV DNA detection in patients with SLE and healthy controls.

EBV DNA Status	SLE, *n* (%)	HC, *n* (%)
Positive	8 (20.5)	0 (0)
Negative	31 (79.5)	29 (100)

**Table 4:** EBV DNA viral load in EBV DNA-positive SLE samples.

Sample	EBV DNA Copies/mL of Whole Blood
1	1325
2	7682
3	<LLOQ
4	4994
5	<LLOQ
6	60,387
7	12,162
8	12,663

LLOQ, lower limit of quantification, corresponding to 1325 copies/mL of whole blood.

**Table 5:** Clinical and hematological characteristics of patients with SLE according to EBV DNA status.

Characteristic	EBV DNA-Positive (*n* = 8)	EBV DNA-Negative (*n* = 31)	*p*-Value
SLEDAI-2K score, median (IQR)	4 (1.5–6)	2 (0–6)	*p* = 0.8329
Disease duration, months, median (IQR)	9.9 (1.6–25.4)	11.1 (1.5–26.6)	*p* = 0.1978
Anti-dsDNA, IU/mL, median (IQR)	62.5 (5–242.5)	36.5 (4.8–95.8)	*p* = 0.4475
At disease onset, *n* (%)	1 (12.5)	7 (22.6)	*p* > 0.9999
Ongoing treatment, *n* (%)	5 (62.5)	25 (80.6)	*p* = 0.3548
White blood cell count, cells/µL, median (IQR)	7040 (4970–8380)	7040 (5307–8380)	*p* = 0.4035
Neutrophil count, cells/µL, median (IQR)	4150 (2690–5490)	3890 (2562–5490)	*p* = 0.9069
Lymphocyte count, cells/µL, median (IQR)	1670 (870–2520)	2115 (985–2600)	*p* = 0.0727

Continuous variables were compared using the Mann–Whitney U test, whereas categorical variables were compared using Fisher’s exact test. Data are presented as median (IQR) or *n* (%), as appropriate. SLEDAI-2K = Systemic Lupus Erythematosus Disease Activity Index 2000; EBV = Epstein–Barr virus; WBCs = white blood cells; IQR = interquartile range. No statistically significant differences were observed.

## Data Availability

The data presented in this study are available on request from the corresponding author. The data are not publicly available due to privacy restrictions.

## References

[1] Robinson W.H., Younis S., Love Z.Z., Steinman L., Lanz T.V. (2024). Epstein-Barr virus as a potentiator of autoimmune diseases. Nat. Rev. Rheumatol..

[2] Young L.S., Rickinson A.B. (2004). Epstein-Barr virus: 40 years on. Nat. Rev. Cancer.

[3] Thorley-Lawson D.A. (2001). Epstein-Barr virus: Exploiting the immune system. Nat. Rev. Immunol..

[4] Kuppers R. (2003). B cells under influence: Transformation of B cells by Epstein-Barr virus. Nat. Rev. Immunol..

[5] Jog N.R., James J.A. (2020). Epstein Barr virus and autoimmune responses in systemic lupus erythematosus. Front. Immunol..

[6] Lossius A., Johansen J.N., Torkildsen O., Vartdal F., Holmoy T. (2012). Epstein-Barr virus in systemic lupus erythematosus, rheumatoid arthritis and multiple sclerosis: Association and causation. Viruses.

[7] Draborg A.H., Duus K., Houen G. (2013). Epstein-Barr virus in systemic autoimmune diseases. Clin. Dev. Immunol..

[8] Pender M.P. (2003). Infection of autoreactive B lymphocytes with EBV, causing chronic autoimmune diseases. Trends Immunol..

[9] Draborg A.H., Izarzugaza J.M.G., Houen G. (2016). How compelling are the data for Epstein-Barr virus being a trigger for systemic lupus and other autoimmune diseases?. Curr. Opin. Rheumatol..

[10] Tsokos G.C. (2011). Systemic lupus erythematosus. N. Engl. J. Med..

[11] Kaul A., Gordon C., Crow M.K., Touma Z., Urowitz M.B., van Vollenhoven R., Ruiz-Irastorza G., Hughes G. (2016). Systemic lupus erythematosus. Nat. Rev. Dis. Primers.

[12] James J.A., Neas B.R., Moser K.L., Hall T., Bruner G.R., Sestak A.L., Harley J.B. (2001). Systemic lupus erythematosus in adults is associated with previous Epstein-Barr virus exposure. Arthritis Rheum..

[13] James J.A., Kaufman K.M., Farris A.D., Taylor-Albert E., Lehman T.J., Harley J.B. (1997). An increased prevalence of Epstein-Barr virus infection in young patients suggests a possible etiology for systemic lupus erythematosus. J. Clin. Investig..

[14] Larsen M., Sauce D., Deback C., Arnaud L., Mathian A., Miyara M., Boutolleau D., Parizot C., Dorgham K., Papagno L. (2011). Exhausted cytotoxic control of Epstein-Barr virus in human lupus. PLoS Pathog..

[15] Yu S.F., Wu H.C., Tsai W.C., Yen J.H., Chiang W., Yuo C.Y., Lu S.N., Chiang L.C., Hsieh S.C., Chen J.Y. (2005). Detecting Epstein-Barr virus DNA from peripheral blood mononuclear cells in adult patients with systemic lupus erythematosus in Taiwan. Med. Microbiol. Immunol..

[16] Moon U.Y., Park S.J., Oh S.T., Kim W.U., Park S.H., Lee S.H., Cho C.S., Kim H.Y., Lee W.K., Lee S.K. (2004). Patients with systemic lupus erythematosus have abnormally elevated Epstein-Barr virus load in blood. Arthritis Res. Ther..

[17] Kang I., Quan T., Nolasco H., Park S.H., Hong M.S., Crouch J., Pamer E.G., Howe J.G., Craft J. (2004). Defective control of latent Epstein-Barr virus infection in systemic lupus erythematosus. J. Immunol..

[18] Jog N.R., Young K.A., Munroe M.E., Harmon M.T., Guthridge J.M., Kelly J.A., Kamen D.L., Gilkeson G.S., Weisman M.H., Karp D.R. (2019). Association of Epstein-Barr virus serological reactivation with transitioning to systemic lupus erythematosus in at-risk individuals. Ann. Rheum. Dis..

[19] Banko A., Cirkovic A., Miskovic R., Jeremic I., Grk M., Basaric M., Lazarevic I., Raskovic S., Despotovic A., Miljanovic D. (2023). Epstein-Barr virus infection as potential indicator of the occurrence and clinical presentation of systemic lupus erythematosus. Front. Immunol..

[20] McClain M.T., Heinlen L.D., Dennis G.J., Roebuck J., Harley J.B., James J.A. (2005). Early events in lupus humoral autoimmunity suggest initiation through molecular mimicry. Nat. Med..

[21] Poole B.D., Scofield R.H., Harley J.B., James J.A. (2006). Epstein-Barr virus and molecular mimicry in systemic lupus erythematosus. Autoimmunity.

[22] Cusick M.F., Libbey J.E., Fujinami R.S. (2012). Molecular mimicry as a mechanism of autoimmune disease. Clin. Rev. Allergy Immunol..

[23] Harley J.B., Chen X., Pujato M., Miller D., Maddox A., Forney C., Magnusen A.F., Lynch A., Chetal K., Yukawa M. (2018). Transcription factors operate across disease loci, with EBNA2 implicated in autoimmunity. Nat. Genet..

[24] Akutsu Y., Ota M., Itamiya T., Mori M., Morio T., Yamamoto K., Okamura T., Fujio K. (2025). Effect of Epstein-Barr Virus infection on gene regulation in immune cells of patients with immune-mediated diseases. J. Autoimmun..

[25] Younis S., Love Z.Z., Hoh R.A., Robinson W.H., Steinman L., Lanz T.V. (2025). Epstein-Barr virus reprograms autoreactive B cells as antigen-presenting cells in systemic lupus erythematosus. Sci. Transl. Med..

[26] Munroe M.E., Anderson J.R., Gross T.F., Stunz L.L., Bishop G.A., James J.A. (2021). Epstein-Barr functional mimicry: Pathogenicity of oncogenic latent membrane protein-1 in systemic lupus erythematosus and autoimmunity. Front. Immunol..

[27] Zhang X., Hu S., Luo P., Li Z., Chen Z., Xia C., Fan L., Li R., Chen H. (2025). The regulatory effect and molecular mechanism of Epstein-Barr virus protein LMP-1 in SLE susceptibility gene expression. Immunol. Lett..

[28] Eliopoulos A.G., Young L.S. (2001). LMP1 structure and signal transduction. Semin. Cancer Biol..

[29] Hayden M.S., Ghosh S. (2008). Shared principles in NF-κB signaling. Cell.

[30] Kieser A., Sterz K.R. (2015). The latent membrane protein 1 (LMP1). Curr. Top. Microbiol. Immunol..

[31] Moore K.W., de Waal Malefyt R., Coffman R.L., O’Garra A. (2001). Interleukin-10 and the interleukin-10 receptor. Annu. Rev. Immunol..

[32] Saraiva M., O’Garra A. (2010). The regulation of IL-10 production by immune cells. Nat. Rev. Immunol..

[33] Akira S., Takeda K. (2004). Toll-like receptor signalling. Nat. Rev. Immunol..

[34] Alexopoulou L., Holt A.C., Medzhitov R., Flavell R.A. (2001). Recognition of double-stranded RNA and activation of NF-kappaB by Toll-like receptor 3. Nature.

[35] Munoz L.E., Lauber K., Schiller M., Manfredi A.A., Herrmann M. (2010). The role of defective clearance of apoptotic cells in systemic autoimmunity. Nat. Rev. Rheumatol..

[36] Cory S., Adams J.M. (2002). The Bcl2 family: Regulators of the cellular life-or-death switch. Nat. Rev. Cancer.

[37] Youle R.J., Strasser A. (2008). The BCL-2 protein family: Opposing activities that mediate cell death. Nat. Rev. Mol. Cell Biol..

[38] Elmore S. (2007). Apoptosis: A review of programmed cell death. Toxicol. Pathol..

[39] Piao Y., Li L., An R., Cui D., Cui X., Jiang L., Jin J. (2025). Exploring the Link Between Genetic Predictors of Systemic Lupus Erythematosus and Epstein-Barr Virus Infections. Int. J. Rheum. Dis..

